# In Vitro and In Vivo Activities of Tilmicosin and Acetylisovaleryltylosin Tartrate against *Toxoplasma gondii*

**DOI:** 10.3390/ijms23179586

**Published:** 2022-08-24

**Authors:** Yazhen Ma, Xinru Cao, Hui Wang, Xingju Song, Dandan Hu

**Affiliations:** Key Laboratory of Prevention and Control for Animal Disease, College of Animal Science & Technology, Guangxi University, Nanning 530004, China

**Keywords:** *Toxoplasma gondii*, tilmicosin, acetylisovaleryltylosin tartrate, drug, toxoplasmosis

## Abstract

*Toxoplasma gondii* is a widespread intracellular pathogen that infects humans and a variety of animals. The current therapeutic strategy for human toxoplasmosis is a combination of sulphadiazine and pyrimethamine. However, this combination still has a high failure rate and is ineffective against chronic infections. Therefore, it is important to discover a new anti-*T. gondii* drug that is safer and more effective in both humans and animals. In this study, we describe the anti-*T. gondii* activities of the 16-membered macrolide tilmicosin and acetylisovaleryltylosin tartrate (ATLL). Both tilmicosin and ATLL potently inhibited *T. gondii* with a half-maximal effective concentration (EC_50_) of 17.96 μM and 10.67 μM, respectively. Interestingly, tilmicosin and ATLL had different effects on the parasites. ATLL exhibited a potent inhibitory effect on intracellular parasite growth, while tilmicosin suppressed parasites extracellularly. By studying the lytic cycle of *T. gondii* after treatment, we found that ATLL potently inhibited the intracellular proliferation of tachyzoites, while tilmicosin affected the invasion of tachyzoites. Immunofluorescence analysis using ATLL-treated *T. gondii* showed morphologically abnormal parasites, which may be due to the inhibition of tachyzoite proliferation and division. In addition, tilmicosin and ATLL significantly delayed the death of mice caused by acute toxoplasmosis. Our results suggest that ATLL has potent anti-*Toxoplasma* activity both in vitro and in vivo and may be an alternative to toxoplasmosis in the future.

## 1. Introduction

As an obligate intracellular apicomplexan parasite, *Toxoplasma gondii* infects almost all warm-blooded animals, including one-third of the human population. It causes severe toxoplasmosis in pregnant women and immunocompromised individuals worldwide [1,2,3]. Humans can become infected by ingestion of oocyst-contaminated water, vegetables and fruits and/or ingestion of tissue cysts in undercooked meat [1]. Ingested parasites can be transformed into tachyzoites and spread throughout the body. As the body’s immune response builds up, the parasites develop into tissue cysts and the infection proceeds to the incubation stage (chronic infection) [4]. When cysts are in immunosuppressed or immunocompromised individuals, bradyzoites are egressed from the cysts and transformed into tachyzoites, causing acute infection [5]. In addition to humans, many animals (pigs, cattle, sheep, horses, dogs, cats, chickens, etc.) are infected with *T. gondii* at a high rate. Serological surveys have shown that 30% of pigs worldwide are infected with *T. gondii*, and the rate of *T. gondii* infection in some areas of China is even as high as 70% [6]. When acute toxoplasmosis occurs in pig farms, the morbidity rate can reach 100% and the mortality rate can be over 60% [7].

Currently, the effective treatment for human toxoplasmosis is a combination of sulphadiazine and pyrimethamine [8]. However, this combination still has a high failure rate and is ineffective against chronic infections. Moreover, different severe complications such as teratogenic potential, reversible myelosuppression, neutropenia, thrombocytopenia, hypersensitivity reactions and hepatic necrosis have been reported [9,10,11,12,13]. In addition, other drugs, such as azithromycin, clarithromycin, spiramycin, atovaquone, and cotrimoxazole (trimethoprim-sulfamethoxazole), have been used for clinical toxoplasmosis. However, no treatment has been found to be more effective than conventional treatment [14,15,16]. Therefore, it is important to develop a new anti-*T. gondii* drug that is safer and more effective for both humans and animals.

Tilmicosin and acetylisovaleryltylosin tartrate (ATLL) are both derivatives of the 16-membered macrolide tylosin, which are macrolide antibiotics commonly used in livestock and poultry for bacterial infections [17,18]. Compared with 14-membered macrolide antibiotics, 16-membered macrolides exhibit some preferable advantages, such as gastrointestinal tolerability, structural flexibility, and lack of inducible resistance [2]. The development of new 16-membered macrolides is a hot topic for researchers [17]. ATLL, which is generated based on tylosin with changes in 3-acetyl-40-isovaleryl group, is a new broad-spectrum third-generation macrolide antibiotic with a 16-membered lactone ring [19]. Tilmicosin is referred to as a semi-synthetic macrolide extracted from the fermentation products of *Streptomyces fradiae* [20]. Previous studies have shown that tylosin A and tylosin B are active against the malaria parasite *Plasmodium falciparum*, which made us wander whether the upgraded macrolides have inhibitory effects against another Apicomplexan parasite *T. gondii* [21].

Therefore, in order to test the anti-*Toxoplasma* activities of tilmicosin and ATLL, parasite invasion, proliferation and their morphology were examined after drug treatment. A mouse model of *T. gondii* acute infection was also established to evaluate the in vivo effects of tilmicosin and ATLL. Our results indicate that both tilmicosin and ATLL have potent anti-*Toxoplasma* activity but with divergent parasite killing mechanisms, and our data will inform the development of future anti-toxoplasmosis drugs.

## 2. Results

### 2.1. Cytotoxicity Activity of Tilmicosin and ATLL

**Figure 1:**
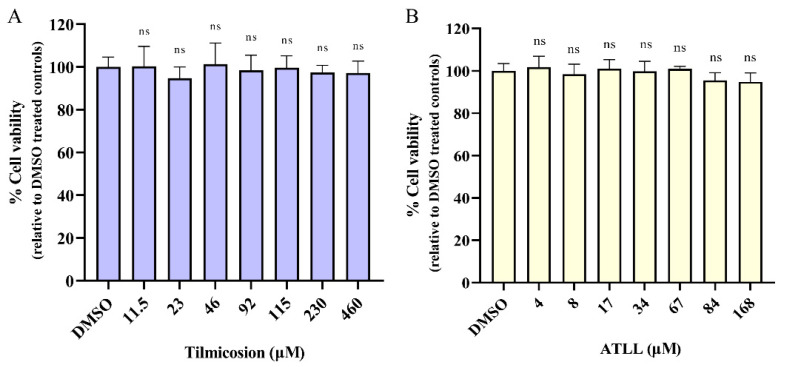
Cytotoxic concentrations of tilmicosin and ATLL. Different concentrations of tilmicosin (**A**) or ATLL (**B**) were treated on Vero cells for 24 h, and then cytotoxicity was determined using CCK-8 reagent. The absorbance was measured at 450 nm. Data are expressed as mean ± SD in triplicate. ns = not significant, indicating statistically insignificant results, as determined by *t*-test.

CCK-8 assay was performed to detect the effects of tilmicosin and ATLL on the viability of Vero cells. The results showed that tilmicosin had no effect on the growth of Vero cells at concentrations below 460 μM (Figure 1A), while ATLL had no effect on the growth of Vero cells at concentrations below 168 μM (Figure 1B). These indicate that ATLL and tilmicosin are not toxic to host cells at high concentrations compared to the control group.

### 2.2. Tilmicosin and ATLL Have Good Anti-T. gondii Effect In Vitro

**Figure 2:**
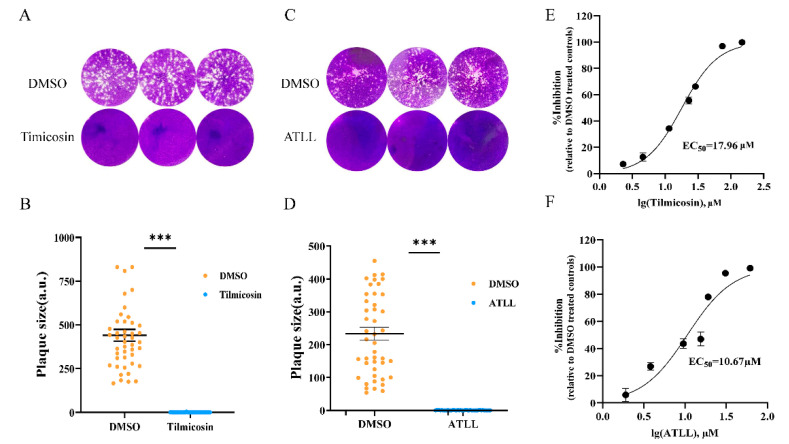
Potent inhibition of *T. gondii* growth by tilmicosin and ATLL. (**A**) Comparison of tilmicosin- or DMSO-treated parasite growth for plaque assays. Each well was infected with 200 parasites and grown for 7 d in the absence or presence of 23 µM tilmicosin. (**B**) Calculation of plaque areas by randomly selecting at least 20 plaques and measuring them using pixel points in Photoshop C6S software (Adobe, San Jose, CA, USA). Data were compiled from three independent experiments. (**C**) Comparison of plaque assays for parasite growth with 17 µM ATLL or DMSO treatment. (**D**) Statistics of plaque area after ATLL treatment. (**E**,**F**) Demonstration of a dose-dependent inhibitory effect of Tilmicosin or ATLL on *T. gondii*. Parasites were allowed to infect Vero cells for 24 h, then compound dilutions were added and incubated for 24 h. The EC_50_ was calculated using the log (inhibitor) versus response-variable slope (four-parameter) regression equation in GraphPad Prism 8 (GraphPad, La Jolla, CA, USA). Means ± standard deviations (SD) are shown for the results of three independent experiments, each condition containing three replicates. The EC_50_ for tilmicosin and ATLL was 17.96 µM (95% CI, 16.58 µM to 19.40 µM) and 10.67 µM (95% CI, 8.684 µM to 12.84 µM), respectively. Asterisks indicate statistically significance. *** *p* < 0.001.

To evaluate the growth of *T. gondii* RH strain tachyzoites treated with tilmicosin or ATLL, the plaque assay was performed. The results showed that there was no plaque formation after tilmicosin or ATLL treatment, which was significantly different from the DMSO groups (Figure 2A–D). To determine the effect of different concentrations of tilmicosin or ATLL on tachyzoites, the luciferase expressing *T. gondii* RH strain (TgRH-Luc) was used to detect reduced luciferase activity. Parasites in Vero cells were treated with different concentrations of tilmicosin or ATLL in an in vitro drug inhibition assay. The half-maximal effective concentrations (EC_50_) of tilmicosin or ATLL were calculated as 17.96 μM (95% confidence interval [CI], 16.58 to 19.40 μM) and 10.67 μM (95% confidence interval [CI], 8.68 to 12.84 μM) (Figure 2E,F), respectively. The results indicated that tilmicosin and ATLL had good inhibitory effects on *T. gondii* tachyzoites in a dose-dependent manner.

**Figure 3:**
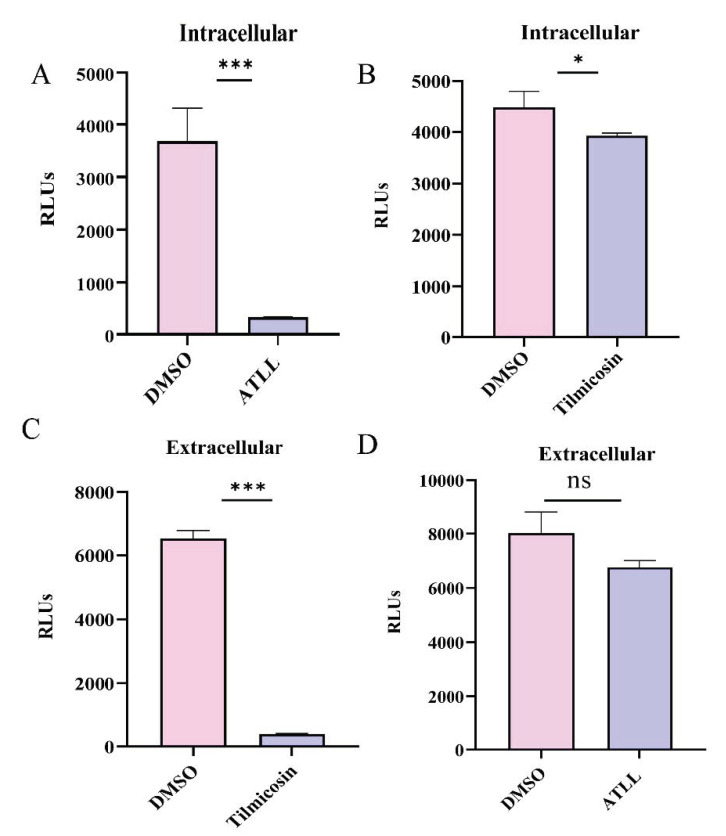
Differential effects of tilmicosin and ATLL on intracellular and extracellular tachyzoites. Vero cells were infected with 1 × 10^5^ TgRH-Luc parasites for 1 h and then cultured with tilmicosin (**A**) or ATLL (**B**). After 24 h, cells and parasites were lysed and the relative luminescence (RLUs) were measured. Parasites were pretreated extracellularly with tilmicosin (35.92 μM) (**C**) or ATLL (21.34 μM) (**D**) for 3 h, then centrifuged to remove the drug and inoculated onto Vero cells in 96-well plates. After 24 h, cells and parasites were lysed and the RLUs were measured. Experiments were repeated three times. The intracellular and extracellular RLUs of parasites after treatment with tilmicosin or ATLL indicates TgRH-Luc activity. These values represent means ± SD (n = 3). Asterisks indicate statistically significant results. ns = not significant, *** *p* < 0.001, and * *p* < 0.05.

The intracellular and extracellular antiparasitic effects of tilmicosin (35.92 μM) and ATLL (21.34 μM) were evaluated at 2-fold concentration of EC_50_. ATLL significantly inhibited the growth of intracellular parasites compared to the control group (Figure 3A). Tilmicosin also exhibited some inhibitory effect, but a significant number of parasites still survived compared to the ATLL group (Figure 3B). The inhibitory effect of ATLL was much better than that of tilmicosin intracellularly. However, the inhibitory effect of tilmicosin and ATLL on extracellular parasites was completely opposite to that of intracellular parasites. Tilmicosin significantly reduced the survival of extracellular parasites, while ATLL did not (Figure 3C,D).

### 2.3. Differential Effects of Tilmicosin and ATLL on Tachyzoite Invasion and Proliferation

**Figure 4:**
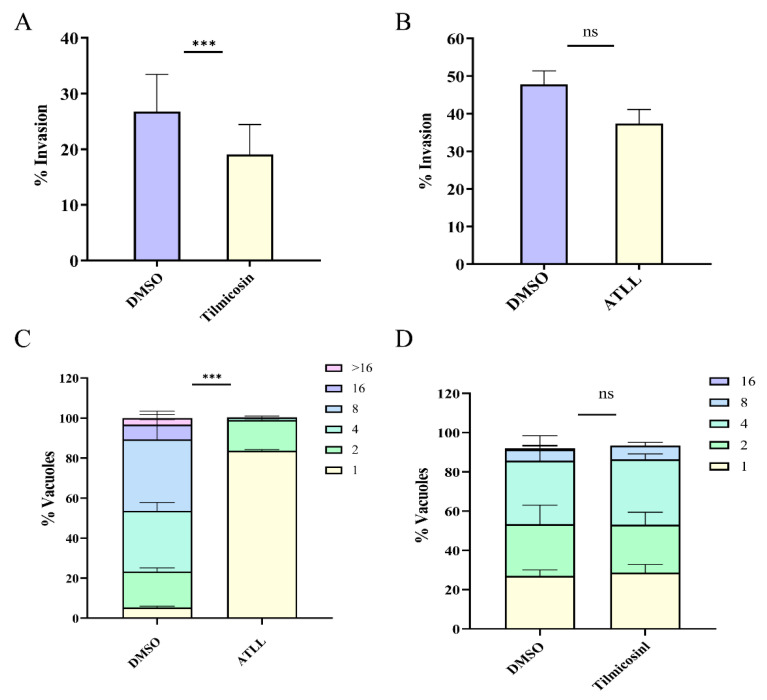
Differential effects of tilmicosin and ATLL on tachyzoite invasion and proliferation. 1 × 10^5^ parasites were inoculated onto HFF cells in 12-well plates and cultured for 24 h with 26.94 μM tilmicosin (**A**) or 16 μM ATLL (**B**). DMSO was used as a control. IFA was performed with anti-TgGAP45 antibody and Hoechst. Invasion rates of parasites treated with different fractions were based on the number of parasite-infected cells divided by the total number of cells in one horizon. Asterisks indicate statistically significant results (*p* < 0.05 as determined by *t*-test). Data are means ± SD (error bars) of three independent experiments. Intracellular replications of parasites were determined by 26.94 μM tilmicosin (**C**) or 16 μM ATLL (**D**) treatment. The experiments were compiled from three separate assays, and in each assay, 100 total PVs of each strain were counted. Data were determined by two-way ANOVA analysis. Asterisks indicate statistically significance. ns = not significant, and *** *p* < 0.001.

The lytic cycle of *T. gondii* tachyzoites in host cells can be divided into five steps: attachment, invasion, vacuole formation, replication, and egress [22]. The reduction in plaque formation may be caused by impairment of one or more steps of the lytic cycle. In this study, we evaluated the effects of tilmicosin and ATLL on tachyzoite invasion. The results revealed that the invasion rate of parasites treated with tilmicosin (26.94 μM) was 19%, which was significantly lower compared to the DMSO group (*p* < 0.01) (Figure 4A). However, there was no significant difference in the invasion rate of parasites treated with ATLL (16 μM) (Figure 4B). Then, intracellular proliferation was assessed by observing the number of tachyzoites in the vacuoles after tilmicosin and ATLL treatment. The results showed that ATLL potently inhibited the intracellular proliferation of tachyzoites (*p* < 0.01) (Figure 4C). However, tilmicosin did not affect the intracellular proliferation of tachyzoites (Figure 4D). These results suggest different mechanisms for tilmicosin- and ATLL-induced plaque size reduction.

### 2.4. Effect of ATLL on the Morphology and Division of Tachyzoites

**Figure 5:**
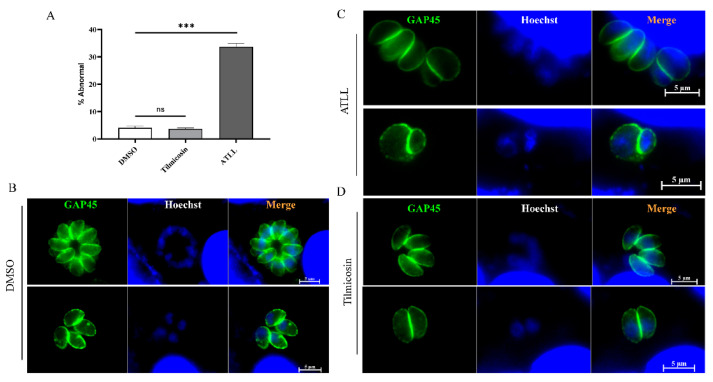
Abnormal morphology of tachyzoites due to ATLL. (**A**) Abnormal division of *T. gondii* after tilmicosin or ATLL treatment. 1 × 10^5^ tachyzoites were used to invade HFF monolayers seeded on cover slips and then treated with DMSO (**B**), ATLL (16 μM) (**C**) and tilmicosin (26.94 μM) (**D**) at 37 °C in 5% CO_2_ for 20 h, respectively. After 24 h post-treatment, the morphology of tachyzoites was observed by IFA using rabbit anti-GAP45 antibody and Hoechst dye. The proportion of vacuoles containing abnormally developed tachyzoites was calculated using fluorescent images of random fields. Statistical analysis was performed by Graph Pad Prism using Student’s *t* test. Asterisks indicate statistically significance. ns = not significant, and *** p < 0.001.

**Figure 6:**
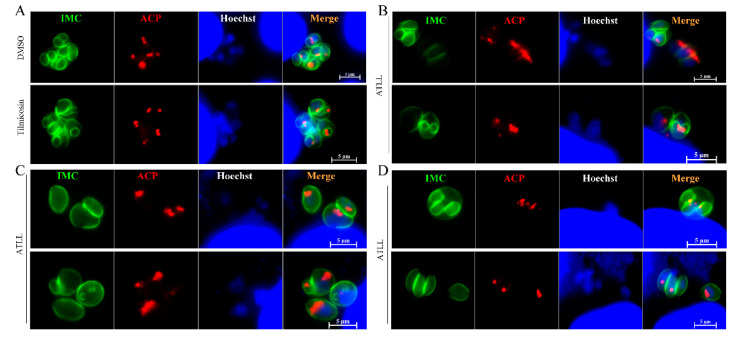
Effects of ATLL on the division of tachyzoites. 1 × 10^5^ tachyzoites were used to invade HFF monolayers seeded on cover slips and then treated with DMSO, tilmicosin (26.94 μM) (**A**) and ATLL (16 μM) (**B**–**D**) at 37 °C in 5% CO_2_ for 20 h, respectively. The division of tachyzoites was observed by IFA using rabbit anti-IMC1 antibody, mouse anti-ACP antibody and Hoechst dye.

Considering the inhibitory effect of tilmicosin and ATLL on parasite growth, we sought to determine whether treatment with tilmicosin and ATLL would alter the morphology and division of tachyzoites. After 20 h of treatment with ATLL, using tachyzoites by immunofluorescence assay (IFA), we found that about 33.7% of tachyzoites in parasitophorous vacuole (PV) were abnormal (Figure 5A). They are mainly round in shape and abnormally divided compared to the control group (Figure 5B,C). After treatment with tilmicosin, the morphology and growth of tachyzoites were the same as in the control group (Figure 5D). To better observe the division, the parasite’s IMC and apicoplast were stained by IFA using specific antibodies. The divisions of IMC and apicoplast were normal in tilmicosin- or DMSO-treated parasites (Figure 6A), but different types of unnatural divisions were observed after ATLL treatment as follows: (1) non-synchronous division (Figure 6B); (2) two parasites from a mother cell with very different sizes (Figure 6C); (3) odd daughter cells formed in a single mother cell (Figure 6D). These results suggest that ATLL has potent activity in disordering the fission of *T. gondii* tachyzoites.

### 2.5. Tilmicosin and ATLL Do Not Affect the Mitochondria and Apicoplast of Tachyzoites

**Figure 7:**
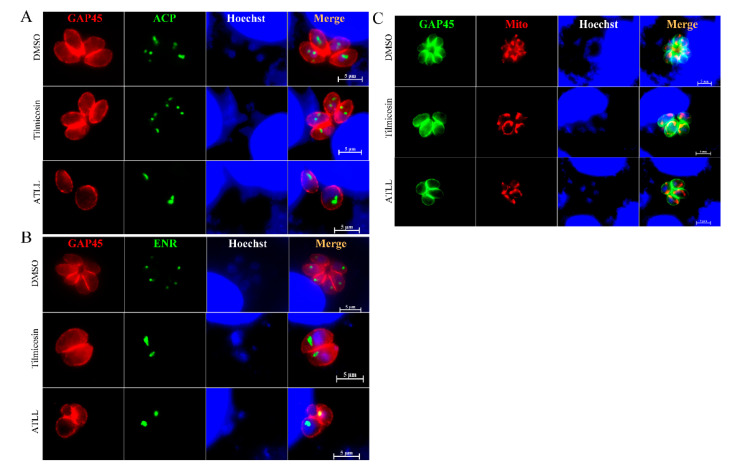
Tilmicosin and ATLL do not affect mitochondria and apicoplast of tachyzoites. (**A**,**B**) 1 × 10^5^ tachyzoites were used to invade HFFs seeded on cover slips and then treated with DMSO, tilmicosin, and ATLL. The apicoplast of the parasites was observed by IFA using specific antibodies against apicoplast proteins (ACP and ENR). Rabbit anti-GAP45 antibody was used as a parasite surface marker (red). In addition, mouse anti-ACP and ENR antibodies were used as an apicoplast marker (green). (**C**) Mitochondria of the parasites were observed using Mito-tracker staining (red). The shapes of the parasites were visualized with anti-GAP45 (green) and nuclear DNA was stained with Hoechst (blue). Scale bar = 5 μm.

It was further investigated whether tilmicosin and ATLL target the mitochondria and apicoplast of *T. gondii*. The expression and localization of apicoplast proteins, enoyl-acyl carrier protein reductase (ENR) and acyl carrier protein (ACP) were observed by IFA. Since the localization of apicoplast proteins ENR and ACP was normal in tilmicosin- and ATLL-treated parasites (Figure 7A,B), tilmicosin and ATLL did not appear to influence apicoplast protein synthesis and trafficking to the apicoplast. Mitochondrial morphology was observed using MitoTracker, and no obvious changes were found between the treatment and control groups, revealing that the morphology and membrane potential of mitochondria are not affected (Figure 7C).

### 2.6. Tilmicosin and ATLL Delay Mortality in Mice Caused by Acute Toxoplasmosis

**Figure 8:**
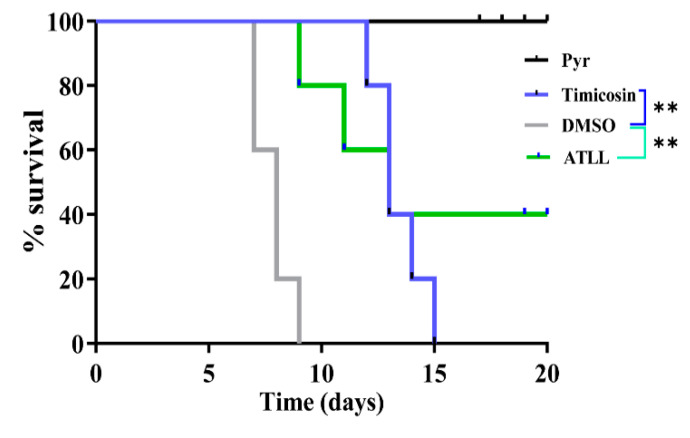
Treatment effects of Tilmicosin and ATLL on acute *T. gondii* infection in mice. Female BALB/c mice (n = 5) were intraperitoneally infected with 500 parasites (type I RH strain). Mice were then treated with intraperitoneal injections of tilmicosin (100 mg/kg/day), ATLL (100 mg/kg/day), pyrimethamine (15 mg/kg/day), or DMSO. The survival of mice was monitored daily. ** *p* < 0.01.

To test the in vivo anti-*Toxoplasma* activity of tilmicosin and ATLL, four groups of BALB/c mice (5 mice/group) were infected intraperitoneally with 500 *T. gondii* tachyzoites (type I RH strain) per group. Each group of mice was treated with daily injections of tilmicosin (100 mg/kg) or ATLL (100 mg/kg) for 20 consecutive days. DMSO was used as a negative control and pyrimethamine (15 mg/kg/day) was treated as a positive control (Figure 8). ATLL treatment resulted in 40% of mice surviving from acute *T. gondii* infection and significantly delayed death. At 15 days post-infection, all mice in the tilmicosin-treatment group died, but it still showed a significant delay in death as all mice in the DMSO-treatment control died at 9 days post-infection. These results suggest that ATLL has better effectiveness than tilmicosin against acute *T. gondii*-infection in vivo. However, the effectiveness of tilmicosin and ATLL on chronic infection should be investigated in future studies.

## 3. Discussion

Global attention has been drawn to *T. gondii* due to the threat it poses to public health and livestock industry. The current treatment for human toxoplasmosis is a combination of sulphadiazine and pyrimethamine [8]. However, therapeutic drugs still have various limitations, such as side effects and drug resistance [9,10,11,12,13]. Thus, alternative treatment options for patients with toxoplasmosis are urgently needed. Several macrolide antibiotics, such as azithromycin, spiramycin, clarithromycin, and tylosin, have been shown to be effective in inhibiting the growth of *T. gondii* both in vitro and in vivo [23]. Spiramycin alone can prevent *Toxoplasma* transmission in early pregnancy [24], while fetuses or newborns have been diagnosed with toxoplasmosis, so spiramycin is discontinued and conventional therapy is administered [12]. In addition, it was mentioned that infants born to mothers treated with spiramycin or pyrimethamine-sulfonamide alone during pregnancy had weaker evidence of clinical manifestations compared to untreated mothers [25].

Tylosin A and tylosin B have been shown to be effective against the malaria parasite *P. falciparum* [21]. In this study, we evaluated and compared the effects of the third-generation macrolide antibiotics tilmicosin and ATLL on *T. gondii* infection in vivo and in vitro. Our results show that both tilmicosin and ATLL have potent anti-*T. gondii* effects, but their mode of action in parasite killing is quite different. Tilmicosin exhibited a strong extracellular parasiticidal effect, whereas ATLL mainly inhibited intracellular parasites. Further studies showed that the EC_50_ of tilmicosin and ATLL were 17.96 μM and 10.67 μM, respectively, similar to those previously reported for spiramycin (17.8 μM) and azithromycin (11.5 μM), and much lower than that of clarithromycin (401 μM) [23,26,27]. In addition, tilmicosin and ATLL had no effect on the growth of cells at high concentrations.

Currently, several macrolide antibiotics have shown prominent inhibitory effects on *T. gondii* in vitro and in vivo and are considered as a possible alternative for the treatment of toxoplasmosis. ATLL and tilmicosin are both derivatives of the 16-membered macrolide tylosin, which inhibit protein synthesis by binding to the 50S subunit of bacterial ribosomes or by affecting the peptidyl transferase reaction within bacteria [17,18]. However, the efficacy and mechanism of ATLL and tilmicosin against *T. gondii* have not been clearly identified. By evaluating the inhibitory effects of tilmicosin and ATLL on *T. gondii* invasion and intracellular proliferation, we were surprised to find that ATLL and tilmicosin have completely different inhibitory effects on the parasites. Treatment with tilmicosin resulted in a significant reduction in the rate of parasite invasion, but did not affect its intracellular replication. Meanwhile, treatment with ATLL resulted in a significant reduction in intracellular replication of the parasites, but did not affect their invasion rate. These results are consistent with the finding that tilmicosin has a strong extracellular parasiticidal effect, while ATLL mainly inhibits intracellular parasites. These results suggest that the reduction in plaque size induced by tilmicosin is mediated by extracellular inhibition of the parasites, which leads to a reduced invasion ability; on the other hand, ATLL inhibits parasite proliferation intracellularly and has no significant effect on extracellular parasites.

To further explore the subcellular target of the drugs, we observed the morphology of the parasites and their organelle structure after treatment using IFA. Compared to the DMSO treatment group, tilmicosin-treated parasites showed normal morphology and divisions, while the ATLL-treated parasites showed rounded morphology and abnormal proliferation. This is consistent with the effects of ATLL and tilmicosin on the invasion and proliferation of the parasites. Interestingly, previous studies have speculated that macrolides may play a role in apicoplast [28]. However, after treating tachyzoites with ATLL or tilmicosin, we found no significant changes in the morphology of apicoplast and mitochondria. Meanwhile, the expression and localization of two functionally completely different apicoplast proteins (ENR and ACP) were observed, and the results showed that their synthesis and localization were not affected, indicating that ATLL and tilmicosin may not be involved in apicoplast and mitochondrial protein synthesis and transport. Based on all the above results, we speculate that although both ATLL and tilmicosin are macrolide antibiotics, the inhibitory mechanisms of these two drugs against *T. gondii* may be different.

Potent in vitro parasite killing, and non-toxicity make ATLL and tilmicosin a potential anti-*Toxoplasma* drug. However, ATLL and tilmicosin had limited therapeutic effect on mortality in acutely infected mice. Tilmicosin prolonged the duration of death in mice, but eventually all mice died. ATLL prolonged mouse death and significantly improved mouse survival, making it an alternative drug. There was still a 60% mortality rate in mice treated with ATLL, indicating the need to combine it with other drugs. In addition, the effectiveness of tilmicosin and ATLL on tissue cysts (chronic infection) should be investigated in future studies.

## 4. Materials and Methods

### 4.1. Ethical Statement

Four-week-old BALB/c mice were administrated in accordance with the recommendations of the Guide for the Care and Use of Laboratory Animals of the Ministry of Science and Technology of China. All experimental procedures were approved by the Institutional Animal Care and Use Committee of Guangxi University (approval number: Gxu-2021-012).

### 4.2. Parasites and Cell Culture

Human foreskin fibroblasts (HFFs) and green monkey kidney cells (Vero) were purchased from the ATCC (Manassas, VA, USA) and cultured in Dulbecco’s Modified Eagle Medium (DMEM) supplemented with 10% fetal bovine serum. The *T. gondii* type I RH strain expressing firefly luciferase (TgRH-Luc) was used, resuscitated from liquid nitrogen and transferred to Vero cells. Parasites were maintained in Vero cells in DMEM supplemented with 2% fetal bovine serum at 37 ℃ and 5% CO_2_.

### 4.3. Compounds

A stock solution (20 mg/mL) of tilmicosin and ATLL (Shanghai Macklin Biochemical Co., Ltd., Shanghai, China) was prepared in 100% dimethyl sulfoxide (DMSO), aliquoted and kept at −20 °C until use.

### 4.4. Cytotoxicity Assay

The cytotoxicity of tilmicosin or ATLL was evaluated in the Vero cell line using a CCK-8 reagent. Briefly, Vero cells (5000 cells/well) were cultured in 96-well plates at 37 °C and 5% CO_2_ for 24 h. Different concentrations of tilmicosin (11.5–460 μM) and ATLL (4–168 μM) were then treated on Vero cells for 24 h, and cytotoxicity was subsequently determined using CCK-8 reagent according to the manufacturer’s instructions (Beyotime, Shanghai, China). DMSO was added as a control. Absorbance was measured at 450 nm using a Microplate Absorbance Reader (BioRad, Hercules, CA, USA). Data were compiled from three independent experiments.

### 4.5. Plaque Assay

Cells grown in 12-well plates were infected with 200 parasites and then cultured with tilmicosin (23 μM) or ATLL (17 μM) for 7 days, followed by fixation with PFA and staining with 0.2% crystal violet. The control was treated with DMSO. The plaque area of each strain was counted using Pixel in Photoshop CS6 software (Adobe, San Jose, CA, USA), and data were obtained from three independent experiments.

### 4.6. Immunofluorescence Assays

Immunofluorescence assays were carried out as described previously [29]. Briefly, parasite-infected HFFs were fixed with 4% paraformaldehyde (PFA) and incubated with 0.25% Triton X-100. Samples were then blocked in 3% BSA for 30 min and subsequently incubated with mouse anti-ENR (1:300), rabbit anti-GAP45 antibody (1:300) or mouse anti-ACP (1:300) for 1 h. Then, secondary antibodies conjugated with FITC or Cy3 (1:100) and Hoechst 33258 (Sigma, Burlington, MA, USA) were used for labelling. Images were obtained using fluorescence microscopy (Zeiss, Jena, Germany).

### 4.7. Invasion Assay and Intracellular Replication Assay

The parasites were grown under standard culture conditions for 3 days and forced to egress through a needle. Then, 1 × 10^5^ purified parasites were inoculated into HFF cells in 12-well plates for 24 h with either tilmicosin (35.92 μM) or ATLL (21.34 μM) protocol. The invasion and intracellular replication ability of the parasites were observed and analyzed by immunofluorescence assays using rabbit anti-GAP45 antibody and Hoechst dye under fluorescence microscopy. The intracellular replication rate of the parasite was assessed by counting the number of parasites per vacuole. For the invasion assay, the percentage of invasion was expressed as the number of vacuoles per host cell. Experiments were performed in triplicate.

### 4.8. In Vitro Drug Inhibition Assay for T. gondii

Vero cells were seeded onto 96-well cell plates and cultured at 37 °C in 5% CO_2_ until 100% confluence was achieved. Cells were then infected with 1 × 10^5^ of TgRH-Luc per well. After 24 h, 2-fold serial dilutions of tilmicosin (from 2.3 to 147.27 μM) or ATLL (from 1.68 to 53.67 μM) were treated into each parasite-infected well. Equal amounts of DMSO were used as controls. The relative luminescence units (RLU) of the parasites were detected after 24 h via a fluorescence microplate reader (Tecan, Infinite M200 PRO, Männedorf, Switzerland) using the Bright-Lumi™ II Firefly Luciferase Assay Kit (Beyotime Biotech, Shanghai, China). The percentage of growth inhibition was calculated as: inhibition rate = [(RLU_DMSO_ − RLU_treatment_)/RLU_DMSO_] × 100%. Samples were run in triplicate and three independent assays were performed. Data are expressed as means ± standard deviation (SD). Half-maximal effective concentrations (EC_50_) of compounds and 95% confidence intervals (CI) were extrapolated using the log (inhibitor) versus response-variable slope (four-parameter) regression equation in GraphPad Prism 8 (GraphPad, La Jolla, CA, USA).

### 4.9. T. gondii Intracellular and Extracellular Inhibition Assay

Vero cells were seeded onto 96-well cell plates and cultured at 37 °C in 5% CO_2_ until 100% confluence was achieved. For the intracellular inhibition assay, cells were infected with 1 × 10^5^ TgRH-Luc parasites for 1 h and then cultured with tilmicosin (35.92 μM) or ATLL (21.34 μM) at 37 °C in 5% CO_2_. For the extracellular inhibition assay, extracellular parasites were pretreated with tilmicosin (35.92 μM) or ATLL (21.34 μM) for 3 h, then centrifuged to remove the drug and inoculated on Vero cells in 96-well plates. Cells and parasites were lysed after 24 h and then RLU was measured. Equal amounts of DMSO treatment were used as controls. Experiments were repeated three times.

### 4.10. Parasite Division and Morphological Observation by Immunofluorescence Assays

1 × 10^5^ tachyzoites were used to invade HFF monolayers seeded on cover slips and then treated with tilmicosin (26.94 μM) or ATLL (16 μM) at 37 °C in 5% CO_2_ for 20 h. After 24 h, immunofluorescence assays were performed with mouse anti-ENR (1:300), rabbit anti-GAP45 antibody (1:300) or mouse anti-ACP (1:300) antibody and Hoechst dye to observe the morphology and organelle structure of the parasites. The DMSO treatment group was used as a control.

### 4.11. Therapeutic Effect of Tilmicosin and ATLL on Acute Toxoplasmosis in Mice

Freshly egressed tachyzoites of *T. gondii* RH strain were purified and counted, and then five female BALB/c mice were infected with 500 parasites by intraperitoneal injection (5 mice per group). One day after infection, mice were injected intraperitoneally with tilmicosin (100 mg/kg/day), ATLL (100 mg/kg/day), pyrimethamine (15 mg/kg/day), or DMSO, respectively, for 20 days. Mice were monitored daily for survival.

### 4.12. Statistical Analysis

Graphs and statistical analysis were performed using Graph Pad Prism (San Diego, CA, USA). Graphs represent means and error bars represent standard errors of the means. All data were analyzed using a two-tailed Student’s *t*-test. *p*-values are indicated by asterisks in the figures as follows: * *p* < 0.05; ** *p* < 0.01; *** *p* < 0.001. We consider all *p*-values < 0.05 to be significant.

## 5. Conclusions

In this study, we tested the in vitro and in vivo activities of tilmicosin and ATLL against *T. gondii*. They showed a potent inhibitory effect against *T. gondii* by acting in very different modes. ATLL significantly inhibits the proliferation of intracellular parasites, while tilmicosin suppresses parasites extracellularly by affecting their invasion ability. ATLL treatment also resulted in parasite’s unnatural division and morphology. In addition, tilmicosin and ATLL significantly delayed the death of mice caused by acute *T. gondii* infection. These data provide insight into the knowledge of the different anti-*T. gondii* mechanisms of tilmicosin and ATLL, which will inform the development of future anti-toxoplasmosis drugs.

## Data Availability

All datasets generated for this study are included in the manuscript.
